# An empirical comparison of knowledge and skill in the context of traditional ecological knowledge

**DOI:** 10.1186/1746-4269-9-71

**Published:** 2013-10-16

**Authors:** Eric P Kightley, Victoria Reyes-García, Kathryn Demps, Ruth V Magtanong, Victoria C Ramenzoni, Gayatri Thampy, Maximilien Gueze, John Richard Stepp

**Affiliations:** 1 Department of Applied Mathematics, University of Colorado at Boulder, 526 UCB, Boulder, CO 80309-0526, USA; 2 Department of Anthropology, University of Florida, 1112 Turlington Hall, UF, Gainesville, FL, 32611-7305, USA; 3 ICREA and Institut de Ciéncia i Tecnologia Ambientals, Universitat Autònoma de Barcelona, Edifici C, 08193 Bellatera, Barcelona, Spain; 4 Department of Anthropology, Boise State University, 1910 University Drive, Boise, ID, 83725-1950, USA; 5 Department of Anthropology, Case Western Reserve University, 11220 Bellflower Road, Cleveland, OH, 44106-1712, USA; 6 Department of Anthropology, University of Georgia, 250a Baldwin Hall, Jackson Street, Athens, GA, 30602-1619, USA; 7 Department of Sociology and Anthropology, Denison University, 100 West College Street, Granville, OH, 43023-1100, USA

## Abstract

**Background:**

We test whether traditional ecological knowledge (TEK) about how to make an item predicts a person’s skill at making it among the Tsimane’ (Bolivia). The rationale for this research is that the failure to distinguish between knowledge and skill might account for some of the conflicting results about the relationships between TEK, human health, and economic development.

**Methods:**

We test the association between a commonly-used measure of individual knowledge (cultural consensus analysis) about how to make an arrow or a bag and a measure of individual skill at making these items, using ordinary least-squares regression. The study consists of 43 participants from 3 villages.

**Results:**

We find no association between our measures of knowledge and skill (core model, *p* > 0.5, **R**^**2**^ = .132).

**Conclusions:**

While we cannot rule out the possibility of a real association between these phenomena, we interpret our findings as support for the claim that researchers should distinguish between methods to measure knowledge and skill when studying trends in TEK.

## Background

Traditional ecological knowledge refers to people’s knowledge, practices, and beliefs about the relationships between organisms and their environment [1]. Many researchers are concerned that this knowledge is being lost and that this loss may have negative consequences for human health [2]; however, research on the relationship between TEK and economic development has returned inconsistent results. Some studies find a negative association between economic development and TEK [3-6], but others find no association [7-9] or even a positive association [10], and still others find that the direction of the association depends upon the measures used [11-13].

Studies analyzing the association between TEK and health, limited thus far to horticulturalists in the Bolivian Amazon, show a relatively more consistent positive association, but the magnitude of the association is small. Knowledge of useful plants is associated with nutritional status [14] and with several indices of children’s health [15], but knowledge of edible and medicinal plants is not associated with height (an indicator of health and nutritional status) [16].

A possible explanation for the inconsistencies in these trends is that much of the previous research on the associations between TEK and economic development and health does not discriminate between knowledge and the application of knowledge, such as skill or behavior [11,17-19]. For clarity, we refer to this distinction as *knowledge* vs. *skill*. We present arguments from other disciplines identifying the importance of the distinction, and some evidence from TEK research suggesting that the distinction matters here too. We then present data comparing a common measure of TEK that measures knowledge to an instrument we designed to measure the skill of applying this knowledge. The purpose of this comparison is to test the extent to which we can use such measures of knowledge when we really want to be measuring the application of the knowledge.

### Knowledge and skill

Much of a person’s knowledge cannot be articulated explicitly, but is rather "contained in the ability to perform special tasks" [20] (p. 106): it is tacit knowledge [21]. Tacit knowledge includes critical skills like the ability to read people’s faces, or interpret sound patterns in a wood [20]. A large portion of TEK is undoubtedly tacit knowledge (see [5,11,18,22]).

Various ideas related to the concept of tacit knowledge have been articulated, such as Ryle’s differentiation between "knowing-how" and "knowing-that" [23,24] and Russell’s contrasting of knowledge by acquaintance and knowledge by description [25]. Anthropologists often implicitly recognize the difference between knowledge and skill acquisition in children cross-culturally [26]. The epistemological validity of these distinctions is debated [27], but differentiating between knowledge and skill is of practical importance in several fields of research.

For example, scholars in nursing education differentiate between theoretical and practical knowledge [28] and scholars in knowledge management differentiate between explicit and tacit knowledge [29] and articulate and inarticulate knowledge [30]. Researchers in these disciplines study how to measure skill because it is important to be able to evaluate someone’s ability to perform a job. Some of their research suggests that measurement instruments focusing on knowledge fail to measure the skill that is actually needed for employees to perform a job (the skill is tacit). In nursing, for example, performance on course exams (knowledge) does not necessarily predict performance with patients (skill) [31]. Similarly, familiarity with written procedures about software engineering projects (knowledge) does not predict an understanding of how to complete those projects successfully (skill) [32].

### Knowledge and skill in TEK research

The relationship between knowledge and skill is increasingly becoming a topic of research within TEK studies (e.g. [33-35]; see [36] for a review of literature to date). The distinction between knowledge and skill may be important for research that seeks to identify trends in TEK (e.g. [11,18]) or to measure its returns (e.g. [14-16]). The majority of studies investigating trends in TEK exclusively measure knowledge (e.g. [3,4,6-9,12]); for example, by asking people to list plants [4,12] or to identify their names or uses [3,7,9]. Sometimes, however, measures of knowledge may be appropriate only to the extent that they approximate people’s use of that knowledge. Honey collectors in South India may acquire explicit knowledge about bees and honey collecting at young ages, but children are not capable of collecting until they have had time to increase physical stature and practice skills like tree-climbing [37]. One causal explanation offered for the association between TEK and health is that people with more TEK have greater access to nutritional and medicinal plants than people with less TEK [14,15]. These studies use a measure of knowledge and assume that it captures people’s behavior and ability to use that knowledge. The slight magnitude of the associations in these studies might be due to a difference between knowledge and skill.

There is some evidence to address the possibility that measures of knowledge and skill capture different aspects of TEK. Among the Mien in Thailand, for example, people had never used many of the medicinal plants they know of, and had only used a third within the last five years at the time of inquiry [5]. In Madagascar, knowledge of certain uses of the *Dypsis fibrosa* palm is unrelated to the actual use of the palm, which is predicted by other factors, such as village of residence, and many known uses were not employed by anyone reporting them [11]. One study finds that the association between knowledge of plants and the use of these plants depends on the ecosystem from which the plants were derived: people used plants they knew about from the forest more than they did from other environments [22].

In two Tsimane’ villages in the Bolivian Amazon, greater knowledge of plant uses predicts greater use of plants in the more remote village, but this relationship disappears in the village closer to an urban center [17]. Another study with the Tsimane’ compares several measures of knowledge, including agreement with experts about plant uses and knowledge of local ecology, to measures of use, including average plants used and total plants used and total species used, finding that measures of use were only weakly correlated with measures of knowledge [19]. Further research among the Tsimane’ compares individuals’ knowledge about plant uses (knowledge) to their self-reported ability to produce certain items (skill). The two measures are only weakly correlated, and they interact differently with measures of economic development [13].

These findings are consistent with the possibility that there may be a difference between measures of knowledge and of skill. Here, we build on this body of research by refining the measures of knowledge and skill in order to provide a more direct test of the relationship.

## Methods

### The estimation strategy

To test the relationship between knowledge and skill, we compare how much people know about the production of a fishing arrow (for men) or a carrying bag (for women) to their skill at crafting these items among the Tsimane’, an indigenous Amazonian group (see below). Previous research in TEK comparing knowledge to skill has used knowledge about the uses of randomly selected plants to predict an ability to produce items [13]. Here we use a measure of knowledge that is more directly associated with the relevant skill: knowledge of facts about how to produce an item, which we measure by how much an individual agrees with everyone else over a series of questions about the production of the item. Our measure is comparable to those used in other TEK studies (e.g. [10,38-43]). To measure skill, we asked participants to evaluate the quality of traditionally produced items. This measure differs from previous research, which measures skill by self-reporting [13] or by behavior [11].

We fit three models to the data using ordinary least squares (OLS) regression analysis. Each model predicts skill as a function of knowledge, controlling for covariates. We used the following as our core model:

$$S_{\text{ivs}}=\beta_{0}+\beta_{1}K_{\text{ivs}}+\beta_{2}A_{\text{ivs}}+\beta_{3}D_{\text{ivs}}+\varepsilon_{\text{ivs}}$$

where *S*_*ivs*_ is the skill at producing an item of person *i* from village *v* and sex *s*, *K*_*ivs*_ is the person’s knowledge of how to produce the item, *A*_*ivs*_ is the age of the item, *D*_*ivs*_ is a set of dummy variables for a person’s sex and village, and *ε*_*ivs*_ is a normally distributed error term. If the measures of knowledge and skills are associated, then *β*_1_ should be positive and statistically significant.

In the core model (*n* = 43) we include interaction terms for knowledge by sex and by village because measures of TEK often vary according to sex [9] and village of residence [6,40]. We also fit a second model (*n* = 43) excluding these interactions, and a third model excluding the interactions and including women only (*n* = 28), because the low variation in the men’s knowledge scores may influence the results.

### Study location and sample

The Tsimane’ are a horticulturalist and foraging society of ∼8000 people (∼100 villages) in the Bolivian Amazon. Tsimane’ traditional subsistence focused on hunting, fishing, and horticulture [44]. Over the last several decades, the Tsimane’ have been experiencing a process of integration to the market economy that has led them to adopt other economic activities, such as cash crop agriculture, logging, and wage labor [45]. For the last 15 years, the Tsimane’ have been the subjects of a panel study focusing on how integration to the market economy affects their economics, health, and traditional ecological knowledge [8,15,45]. Ethnographic descriptions of the Tsimane’ can also be found in recent books and dissertations [40,46].

We collected data in three villages near the town of San Borja, department of Beni (Bolivia), from June to August 2008. These villages were spread through a continuum of integration to the market economy, defined by material possessions purchased from town, wealth, fluency in Spanish, and several other criteria [45]. A strong predictor of these variables is access to the market town [47], and we selected the three villages for inclusion in the study accordingly, seeking to maximize the variation in economic integration in order to most accurately represent the population. One village was a few hours’ travel by car from San Borja, and thus accessible via minibus, which run on a regular schedule. This village was the most economically integrated. The second village was several hours’ travel upriver by canoe with an outboard motor, and as (at the time of research) most Tsimane’ have access only to canoes without motors, this commute would typically take an entire day round-trip. This village still had regular access to San Borja, but much less frequently. The final village was a several-hour commute by truck down a logging access road. This village had restricted access to San Borja as the only means of commuting consisted of infrequent logging trucks willing to take passengers, and so was the least economically integrated.

Eligible participants consisted of all adults (>18 years of age) who self-identified as household heads. A typical house consisted of a male and female household head, their children, and sometimes their parents (usually when these parents were old enough to require care), and so most houses had two household heads. In each village, all houses were contacted. The study included a total sample size of 43 people: 15 males and 28 females. This discrepancy arose because in the most economically-integrated village, many men had largely abandoned the use of bows and arrows, preferring rifles and fishing rods. There were too few arrows available to obtain a representative sample of the village, and those that were available tended to be old and frequently in disrepair. We therefore excluded the males from the sample in this village.

### Selection of items

We selected items for inclusion in the study and developed questionnaires on the construction of these items. Each participant in the study was given one of the final questionnaires (to measure knowledge) and had her or his item ranked by peers (to measure skill).

To select items for the study, we first used interviews and observations to construct a full inventory of plant-made household items. The list contained 15 items produced by both men and women. We then narrowed the items in the inventory according to the following criteria: 

1. ubiquitous distribution (broadly used)

2. variation in craftsmanship

3. crafted by a single individual

4. important practical function

5. feasibility of borrowing the item for several days

Because Tsimane’ men and women typically craft different items from plants, we selected two sex-specific items: fishing arrows for men and hand-woven carrying bags for women.

### The knowledge variable

Knowledge, defined by information about the production of bows and arrows (men) and the production of traditionally woven bags (women), was measured using responses to a questionnaire on how the item is produced. To construct the questionnaire, we first conducted open-ended interviews with key informants, identified by the community as especially knowledgeable about either bows and arrows or bags. We used the information from open-ended interviews to generate a list of dichotomous (true/false) questions. Questions were pre-tested for comprehension and for variation in answers.

The arrow questionnaire contained questions about whether certain species of plants or birds (for feathers) could be used at particular stages in the process of creating an arrow or a bow. For arrows this included wood for the shaft, feathers for the fletching, plants to make the string used to attach the fletching to the shaft (arrow), and plants to dye the fletching and the string various colors. For bows the questionnaire included wood for the bow and plants for the string. Different types of bows and arrows are used to hunt different game; for example, to shoot fish vs. birds, which allowed for various permutations of these questions. This questionnaire ultimately contained 16 questions. The carrying bag questionnaire contained questions about whether each of eight plants could be used to create a dye of each of four colors (all bags used colored thread). Some colors required mixing multiple plants, and several could be obtained at least two different ways (i.e., using two distinct but possibly overlapping sets of plants). This questionnaire ultimately contained 32 questions.

Ideally, the questionnaires would have been the same length; however, there were roughly double the number of plants involved in the manufacture of dyes as there were in the construction of bows and arrows. We considered three alternative methods: (1) keep the questionnaires as they were, (2) eliminate 16 of the questions from the women’s questionnaire, or (3) add additional questions to the men’s questionnaire. Option (2) would have reduced the volume of data, which was already smaller than ideal (see power analysis in the Results section). Option (3) would have required the addition of questions that were not of the form "can this plant be used for …", differing from the 48 existing questions, and could thus have introduced further bias. For these reasons we chose to proceed with option (1).

### The skill variable

Skill is defined here as the ability to make a useful item and is measured by the quality of an item produced by a participant, as judged by his or her peers. Participants were asked to lend their best fishing arrow (men) or their best hand-woven bag (women). These items were grouped into sets by type of item and village; for example, all of the arrows from village *A* form a set, as do all of the bags from village *A*. We asked all male participants in a village to rank all arrows in a set against each other item, and all female participants to rank all woven bags in a set. Therefore, each male participant who provided an arrow ranked all the arrows from his village and each female who provided a bag ranked all the bags from her village. This approach limited the number of items to be ranked together to a maximum of 10, the upper limit for ranking tasks [48,49]. For the ranking exercise, we took individuals one at a time and presented them with all of the items in their set. We asked them to choose the best one and recorded the answer. Then the best item was removed from the set and the process was repeated with the remaining items until only one item remained (the worst item). Men were asked to choose the best arrow to shoot a fish in the river and women were asked to choose the most well crafted bag. To assess the possibility of personal bias, during pretesting we asked if individuals could identify the maker of a subset of the arrows or bags; no one was able to do so.

### Data analysis

We constructed a measure of knowledge by analyzing responses to the true/false questionnaires using cultural consensus analysis, hereafter CCA [50,51]. This analytical tool is used across the social sciences [52], including TEK research [10,38-43]. CCA works by obtaining a principle axis factoring on person-by-person matrices of responses to a systematic questionnaire, calculating the extent to which variation in responses across people can be reduced to a single factor. The loadings on the first factor indicate individual knowledge; these loadings are termed "competence scores" in the model; we use these values as our *knowledge* variable. When a respondent did not know the answer to a question, a randomly generated value was inserted with a probability of.5 for yes and.5 for no (following [52]). Each questionnaire was analyzed using the formal consensus model [50] in the ANTHROPAC software [53].

We use standard diagnostics to evaluate the goodness-of-fit of the cultural consensus model to the data [52]. To validate the assumption that there is only one set of correct answers, the first and second eigenvalues can be compared. The ratio of the first to second eigenvalue is a measure of the dimensionality of the data; the higher the ratio, the more likely it is that there is only a single pattern (a single set of answers) to the responses. The ratio of the first to the second eigenvalue was 18.46 for the arrow questionnaire and 7.69 for the bag questionnaire, above the conventional threshold of 3:1 used to determine whether consensus exists. A second indicator of the goodness-of-fit of the model to the data is the range and magnitude of the competence scores (first-factor loadings). Competence scores fall between 0 and 1 and the mean should ideally be above.5, indicating that people actually agree about the answers. The mean competence score in our data was.71 for women and.90 for men (Table 1), indicating that there was consensus. Third, to avoid spurious patterns, there should be no more than 10% missing values. In our data, missing values occurred when informants did not know the answer, and account for 6.5% of the data. Fourth, to ensure that there is enough variation in the data, the balance of positive to negative responses in a dichotomous questionnaire should fall between 30% and 70%. Once we exclude the missing values from our data, 91% of the responses to arrow questionnaire and 30% of the responses to the bag questionnaire were positive. Therefore, the ratio for the arrow questionnaire falls outside of the ideal range. It is not clear what effect this bias has on the results of CCA [52].

**Table 1:** Diagnostics and summary statistics for cultural consensus analysis (CCA) results

**Questionnaire**	**Sample**	**Missing**	**Positive**	***λ***_**1**_**/*λ***_**2**_	**Mean**	**Range**
	**size**	**values**	**responses**		**competence (*sd*)**	
Arrow (men)	15	0.9%	91.1%	12.34/0.67 = 18.46	.90 (.12)	.59-.99
Bag (women)	28	9.3%	30.0%	15.23/1.98 = 7.69	.71 (.22)	.29-.96

*Missing values* is the percentage of the responses for which participants declined to respond the question, *Positive Responses* is the percentage of all responses that were affirmative (all questions dichotomous), *λ*_1_/*λ*_2_ is the ratio of the first to second eigenvalues of the CCA analysis.

The knowledge variable is the combination of competence scores for men and for women, with a combined mean (*sd*) of.77 (.21) and a range from.29 to.99. As is typical for competence scores, the values are not normally distributed (Shapiro-Wilk test of normality, *W* = .895, *p* = .0009). Power- and log-transformations did not produce a normal distribution.

To allow comparisons across sets of different sample sizes, the skill variable was generated by dividing the mean rank of each item by the number of items in its set and subtracting this value from one. This transformation produced values with range from 0 (all participants ranked the item as worst) to.9 (all participants ranked the item as best).

To check for meaningful variation in the skill variable, items’ ranks were compared by obtaining a one-way ANOVA on each set of items followed by post-hoc comparisons between each pair of items using Bonferroni’s method (with *α* = .005 to correct for multiple comparisons). Each ANOVA was significant at *p* < .00001 (Table 2) and post-hoc Bonferroni comparisons resulted in two to four groups per set at *p* ≤ .005 (to correct for multiple comparisons, results not shown). The skill variable, transformed from the item rankings, was normally distributed (Shapiro-Wilk normality test, *W* = .9730, *p* > .4) with mean (*sd*) of.44 (.22), and range from.03 to.89 (Table 2).

**Table 2:** Summary statistics for the skill variable

**Group**	***df***_**1**_**,** ***df***_**2**_	***F***	***p* (two-tailed)**	**Mean skill (*sd*)**	**Range**
Village a, Bags	9, 90	21.39	< .0001	.45(.25)	.03-.89
Village b, Bags	9, 90	8.74	< .0001	.45(.21)	.12-.85
Village b, Arrows	7, 56	6.07	< .0001	.44(.20)	.17-.70
Village c, Bags	7, 56	16.72	< .0001	.44(.25)	.13-.72
Village c, Arrows	6, 42	7.21	< .0001	.43(.22)	.14-.69

One-way ANOVA results comparing the ranks assigned to items, with summary statistics. *Skill* is defined to be the transformed mean rank for each item, and includes standard deviation.

In summary, analyses to test the validity of our measures showed that our knowledge variable met all of the assumptions of CCA and passed all diagnostic tests except for balance of answers: 91% of the responses to the arrow questionnaire were positive, which falls outside of the ideal 30-70% range. Additionally, the knowledge variable was not normally distributed. The skill variable represents statistically meaningful variation in quality of items and is normally distributed.

### Ethical approval

This research was approved by the Institutional Review Boards of the University of Florida (#2008-U-650) and Brandeis University (#04217).

## Results

### Testing the model

Knowledge is not significantly associated with skill in any of the models (Table 3), and none of the models is significant (model 1: *F*_6,36_ =.91, *p* > .5; model 2: *F*_5,37_ = 1.17, *p* > .3; model 3: *F*_4,23_ = 1.27, *p* > .3). Therefore, none of our variables predicts skill, and the failure of knowledge to do so is not due to the presence of a covariate.

**Table 3:** Multivariate OLS regressions of skill (outcome variable) against knowledge

	**Model 1**		**Model 2**		**Model 3**	
Explanatory Variable	*β*	*p*	*β*	*p*	*β*	*p*
Knowledge	-.273	.574	-.276	.225	-.287	.286
Sex	-.024	.961	-.038	.666	ˆ	ˆ
Item age	-.005	.090	-.004	.108	-.004	.137
Knowledge*Sex	-.018	.974	ˆ	ˆ	ˆ	ˆ
Knowledge*Village	.028	.906	ˆ	ˆ	ˆ	ˆ
**N**	43		43		28	
**R**^2^	.132		.137		.181	

Regressions contain a constant and a set of binary variables for village (not shown). *β* is the regression coefficient, *p* is the *p*-value. ˆVariable excluded from analysis.

### Robustness

We tested the robustness of our statistical analysis and of our models in several ways. First, we tested the assumptions of OLS regression analysis by performing standard diagnostic tests and by substituting an alternate knowledge variable in an instance where an assumption is violated. Then, we substituted alternatives to several variables to test for methodological biases. None of the changes we made affected the direction, magnitude, or significance of the association. Unless stated, all tests are conducted on model 2.

#### Robustness of statistical analyses

First, we tested three standard assumptions of regression analysis: (1) homoskedasticity (by obtaining a Breusch-Pagan/Cook-Weisberg test for heteroskedasticity), (2) independence of errors (by obtaining variance inflation factors for each predictor variable), and (3) normal distribution of errors (by plotting residual vs. predicted values and by obtaining influence statistics for each value). Results from these tests indicated that the assumptions were not violated.

Second, because the knowledge variable was not normally distributed, we substituted a dichotomized variable for the knowledge variable in model 2. Dichotomizing variables results in a loss of information, but relaxes the normality assumption. The dichotomized variable took a value of 0 for ≤50th percentile and a 1 for >50th percentile of the knowledge score. The substitution did not change the magnitude, direction, or statistical significance of the results.

**Figure 1:**
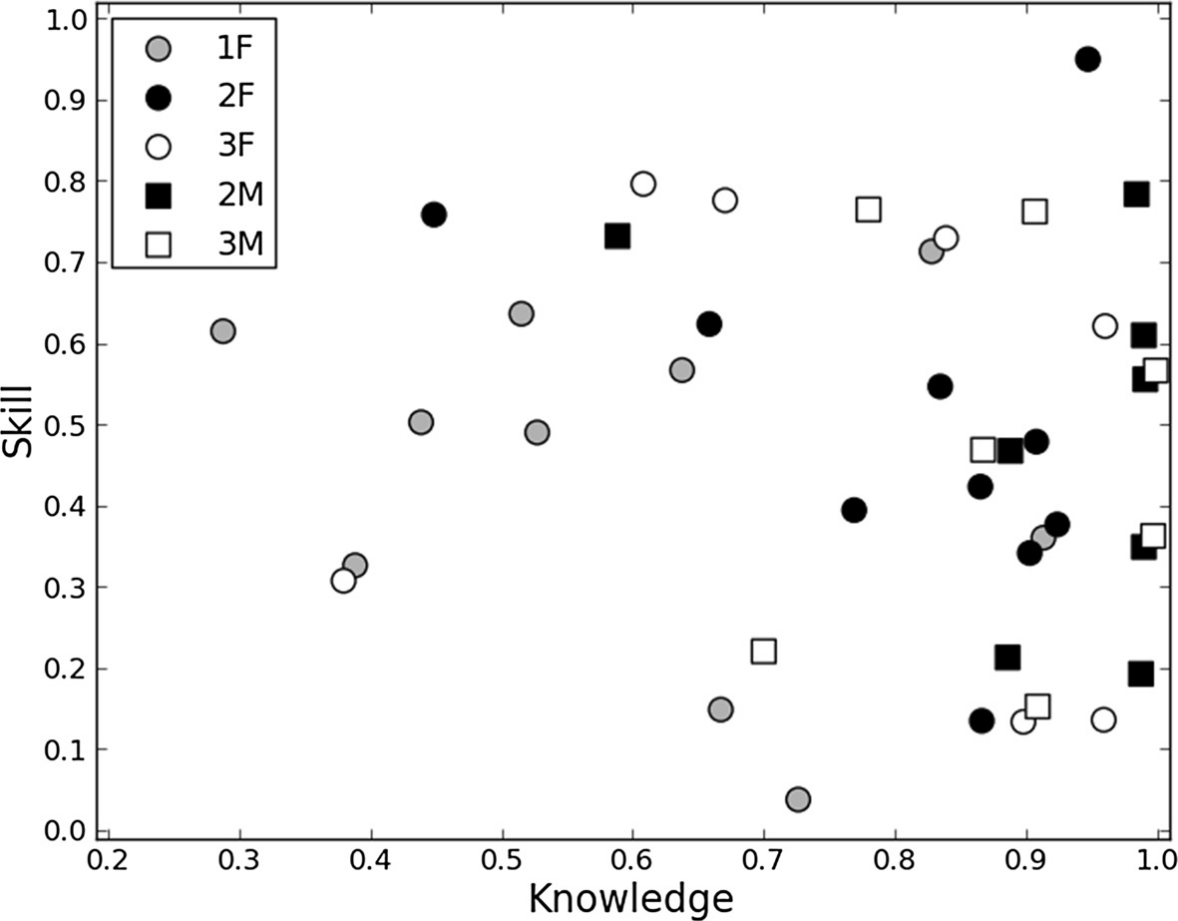
**Individuals’ skill vs. knowledge.** Legend indicates village (number) and gender (Female/Male).

Third, we calculated the sample size required to identify a significant association, given the number of parameters and the correlation coefficient from the regression [54,55]. For model 2, with Type I error rate *α* = .05 and Type II error rate *β* = .2, the calculated sample size should be 87, indicating that there is an increased risk of Type II error (failing to find an association when one really exists). Therefore, we calculated the power of the regression, a measure of the ability of the regression analysis to detect real associations [54,55]. For model 2 the power is.43, far below the conventional threshold of.8. This confirms that the regression analysis is more likely to miss a real association than is conventionally accepted. Lastly, we obtained a scatterplot of skill vs. knowledge by sex and village (Figure 1) to look for obvious linear or non-linear patterns; none is apparent.

#### Robustness of the models

We made all justifiable adjustments, substitutions, and transformations to challenge the results. First, because knowledge varies by sex and village, it is possible that the knowledge variable used here should be measured by how much a person agrees with informants from their village or with the entire sample, instead of how much they agree with informants from their own sex. We substituted knowledge variables measuring (a) agreement with own village and (b) agreement with the entire sample, instead of with own sex. This was accomplished by applying the CCA procedure to different subsets of responses: (a) each village separately and (b) the entire sample (all participants took the male and female questionnaire).

Second, our measure of knowledge is based on agreement between individuals. A common alternative to this is to count the number of plants/birds identified as useful [5,11]. In our questionnaire, a positive response indicates that the particular species of bird or plant can be used for a certain task, and a negative response that the species cannot. Therefore, we substituted the number of species that each respondent said was useful for the knowledge variable.

Lastly, because skill for females depends upon the material used to make the bag (two-sample *t*-test, *df* = 26, *t* = 2.53, *p* = .0179), we fit model 3 with an additional binary variable for material (0= contains wool, 1= does not contain wool).

## Discussion

We found no statistically significant association between our measures of knowledge and skill. There are several possible interpretations of this result, which fall under two categories: it is possible (1) that knowledge and skill are actually distinct, conceptually and/or methodologically, and it is possible (2) that the data are inadequate, because of a small sample size and/or inappropriate variables. We discuss the rationale for, and implications of, these potential explanations. While we cannot exclude the possibility of a real association between knowledge and skill, it is clear that our two methods do not measure the same thing, and we argue that this finding is applicable to other studies in TEK. We conclude that, for research on trends in TEK or its returns, the choice of a method to measure TEK should be justified against the alternative methods available. This justification should include explicit a priori reasons to expect an association between the TEK variable and its hypothesized covariates.

### Knowledge and skill may be different

We measured how much people know about how to make an item and how good they were at making the same item. We did not find any association between the two variables. One interpretation of this result is that measures of knowledge and skill in TEK research may not measure the same thing. We designed our measures of knowledge and skill in an attempt to maximize the association between them; nevertheless, there are very good reasons to expect that these two measures ought not to be associated, as discussed in the Limitations section below. For example, the characterstics used to judge the quality of an item appeared to have nothing to do (at least not directly) with the species used to construct the item. It is therefore possible that we are measuring two different areas of knowledge, as opposed to contrasting measures of knowledge and skill pertaining to the same underlying core of knowledge. It could thus very well be the case that in this work, we were mistaken to expect our two measures to be related in the first place. All the more reason to make sure we are measuring what we want to be measuring! The conclusion we draw from the lack of association between our measures of knowledge and of skill is that measuring different aspects of TEK can return different results.

This conclusion is consistent with several TEK studies finding that measures of knowledge and skill or behavior are not necessarily associated [5,11,17,22]. Previous research with the Tsimane’, however, has found that measures of knowledge and measures of self-reported skill are positively associated, though weakly [13,19]. The approach to measuring knowledge in these studies is the same that is used here (CCA on plant uses), but the measure of skill is different, defined instead as self-reported ability to make several items from plants.

While the evidence here and elsewhere does not conclusively show that measures of knowledge and skill are different, we find little support for the hypothesis that these measures are interchangeable. We conclude that TEK researchers motivated by a concern that a loss of TEK could have negative consequences for people’s health or economic situations may want to consider using measures of skill in addition to knowledge; at the very least, it ought not be the case that meaures of knowledge (by which we mean measures similar to the knowledge variable used here) are the *de facto* methods of choice. There are many cases in which it is valid to measure knowledge, and rather than claim otherwise, we argue only that such a choice ought to be validated through careful consideration of what we want to measure and why we wish to do so.

### Limitations

We cannot rule out the possibility that, because of the issues discussed below, our analysis failed to identify a real association between knowledge and skill. However, even if more data were to reveal the existence of an association, it appears that it would be slight in magnitude: no obvious pattern presents itself in the scatter plot of knowledge and skill (Figure 1). In fact, to get a positive association, further data would have to reverse an apparently negative trend (*β* = -.2757 for model 2, Table 3).

Our test deviates from ideal conditions in three ways. First, the sample size is smaller (*n* = 43) than the estimated minimum (*n* = 87) given the correlation coefficient. This results in a higher probability of a Type II error, where a real association is missed. Second, the knowledge variable does not follow a normal distribution (regardless of log and power transformations), which violates an assumption of OLS regression. Third, the responses to the arrow questionnaire (males) fall outside of the ideal range of the ratio of positive to negative responses. The effects of this on the results of CCA are unknown [52]; however, the resulting competence scores for men (knowledge variable) had low variation and were right-skewed (mean = .90, *sd* = .12), which is not ideal for regression analysis. For this reason we included model 3, which excludes men from the analysis, though the sample size is even smaller (*n* = 28). We note that all questions generated variation in answers during pretesting, suggesting that the pretest sample was not representative.

There are two potential sources of problems with our knowledge variable: the method itself and our application of it. We used CCA to derive the knowledge variable. A critical assumption of CCA is that greater agreement with everyone else in the sample is assumed to mean greater knowledge about the topic [50,52]. In other words, there is a cultural model from which people draw, and the extent to which people agree with each other reflects the extent to which they draw from this model. This assumption has been challenged [56,57]; sometimes people draw from multiple models [43,58,59], even when the CCA goodness-of-fit diagnostics indicate a single model [60]. This can be a problem for TEK research, for example, in situations where experts have a monopoly on specialized knowledge [43]. However, CCA is commonly used in TEK research [10,38-43], as are several other indices based on agreement (e.g. [61,62]). Any method that bases measures of knowledge on agreement, whether this is agreement with other individuals in the sample, with experts, or with an answer key, may be susceptible to a general form of this issue: we are assuming the existence of, and access to, the "right" answers. This is an unresolved issue in cognitive anthropology in the midst of ongoing debate, and we introduce it only as a potential explanation for our results. We consider this controversy to further substantiate our conclusion that the choice of method to measure TEK must be made carefully.

It is also possible that the content of our questionnaires does not measure the relevant information: maybe knowledge about how to make these items is associated with the skill of making them, and our questions do not capture that knowledge. To develop the questionnaires, we asked several people to describe the process of creating bows, arrows, and bags, and chose questions about steps where there were discrepancies between people’s descriptions. Each of these questions asks which materials can be used at a specific step in the process. It may be that the important variation in knowledge about making these items lies elsewhere (e.g., knowing the angle of curvature of the bow, the speed at which cotton must be spun, etc.). However, we chose our questions to be comparable to those used in other TEK research. The majority of studies measuring knowledge about uses of plants do so using yes/no questions asking whether a particular plant can be used for some purpose (e.g. [5,8,61]). This is an entirely reasonable and common sense approach, but we still do not know if our measure of knowledge captured the relevant knowledge and we cannot test this here. Since our method is comparable to other studies using CCA [10,38-43] and other agreement-based indices [61,62] to measure TEK, we maintain that our findings are relevant.

We developed separate indices of knowledge and skill for men and women, so it is possible that these measures are not comparable and that they should not be combined in an analysis together. This is the consequence of accounting for the difference in TEK between genders [9,11,12,63-66].

Because our specific approach to measuring skill is novel, we cannot compare its performance to previous applications. In conversations with participants, women indicated that they judged the bags by the quality of the pattern (tighter and more intricate weaves were considered better quality), and men indicated that they judged the quality of an arrow primarily by the straightness of the shaft and the balance of weight. The skill variable would be more reliable if we had systematic data on the criteria people used to rank items - do they all use the same criteria? It may be that these criteria are inseparable from each other and from the act of ranking, similar to the Gestalt effect, which maintains that the identification of an image or object is an instantaneous perception that cannot be reduced to the constituent components [67].

Also, we do not know if a higher-ranked item is actually more functional. Furthermore, it may be inappropriate to use the different measures of skill for men and women in the same analysis (as discussed in the knowledge section above). The advantage of our skill variable is that it ought to be more closely related to our knowledge variable than alternative methods, such as frequency of use or self-reported ability to produce an item.

## Conclusion

In this study we compared a measure of knowledge to one of skill. We found no association between these measures. Can we conclude that knowledge and skill are definitely distinct in TEK? No, absolutely not. We cannot even conclude with reasonable confidence that *our specific measures* of knowledge and skill are distinct, in light of the numerous limitations of our study. While we could have done better in retrospect, many of these limitations come with the territory, literally. What we can conclude, however, is that we have certainly not found any support for the claim that these measures are interchangeable. No one has claimed explicitly that they are, but a very specific class of methods (multiple choice questionnaires about plant uses or identitification) is used a majority of the time to measure TEK, sometimes in cases in which the variable of interest can reasonably be described as a person’s ability to use natural resources productively. We argue that sometimes, it might be more appropriate to try to measure this ability more directly, especially in light of the inconsistent results that some of these studies return.

The principal message we wish to communicate is perhaps best illustrated through a thought experiment: we could very well have chosen just one of the two measures we used here and justified this choice; had we chosen the measure of knowledge, we would be in good company. Having done so, we could then have measured some developmental, economic or health-related variable and looked for an association. Supposing we found such an association, we could then have deployed a series of arguments explaining why this result was reasonable. Now, had we chosen the other measure, we could have done everything else exactly as above only to find that there *was no* association. We could then have deployed a different series of arguments explaining why we though *this* result was reasonable. In both cases the arguments could be completely valid: the variables we seek to operationalize are manifestly multifaceted, their complex heterogeneity precluding a simple definition. For example, it is very well possible that certain aspects of development are associated positively with certain aspects of TEK, while others are not; as we have seen, in fact, there is a precedent for this among the Tsimane’ [13,17].

If we could be confident that any reasonably-well justified measure of TEK could substitute for any other, then the preceding hypothetical situation would be of no concern. Of course, no one would think a statement as general as this could be true, but it is frequently the case that we measure knowledge when what we are worried about is people not knowing how to get what they need from natural resources. We tried our best to build two different measures of two potentially different domains (knowledge and skill) that stood as good as chance as we could give them of being correlated, and we found no evidence that they were. We view our particular result here to be another piece in a growing body of literature [5,11,13,17,19,22,37] suggesting that the details of how and why TEK is measured are of the utmost importance. There are many choices to be made; these choices should be justified against alternatives, and this justification should include explicit *a priori* reasons to expect an association between the TEK variable and its hypothesized covariates.

## Competing interests

The authors declare that they have no competing interests.

## Authors’ contributions

All authors contributed to study and method design. EPK, VRG, KD, RVM, VCR, MG, and GT collected data. EPK, VRG, and JRS conducted statistical analyses. EPK prepared the manuscript. All authors read and approved the final manuscript.
